# Exploration of the pathogenesis of polycystic ovary syndrome based on gut microbiota: A review

**DOI:** 10.1097/MD.0000000000036075

**Published:** 2023-12-15

**Authors:** Hua Guo, Jing Luo, Hanmei Lin

**Affiliations:** a Graduate School of Guangxi University of Traditional Chinese Medicine, Nanning City, Guangxi Zhuang Autonomous Region, China; b Department of Gynaecology, The First Affiliated Hospital of Guangxi University of Traditional Chinese Medicine, Nanning City, Guangxi Zhuang Autonomous Region, China.

**Keywords:** gut microbiota, hyperandrogenism, insulin resistance, neuroendocrine dysfunction, polycystic ovary syndrome

## Abstract

Polycystic ovary syndrome (PCOS) is a complex disorde7r influenced by genetic, neuroendocrine, metabolic, environmental, and lifestyle factors. This paper delves into the increasingly recognized role of gut microbiota dysbiosis in the onset and progression of PCOS. Utilizing advances in next-generation sequencing and metabolomics, the research examines the intricate interaction between the gut microbiota and the central nervous system via the gut-brain axis. The paper highlights how disruptions in gut microbiota contribute significantly to PCOS by modulating the release of gut-brain peptides and activating inflammatory pathways. Through such mechanisms, gut microbiota dysbiosis is implicated in hyperandrogenism, insulin resistance, chronic inflammation, and metabolic disorders associated with PCOS. While the relationship between gut microbiota and PCOS has begun to be elucidated, this paper underscores the need for further research to identify specific bacterial strains and their metabolic byproducts as potential therapeutic targets. Therefore, comprehensive studies are urgently needed to understand and fundamentally treat the pathophysiological processes of PCOS, offering valuable insights for future treatment and prevention strategies.

## 1. Introduction

Polycystic ovary syndrome (PCOS) represents one of the most pervasive and intricate endocrinological disorders affecting females within their reproductive years. Affecting an estimated 5% to 10% of this population globally, the prevalence of PCOS shows an alarming annual increase.^[1]^ Beyond its debilitating impact on female fertility, manifesting in symptoms ranging from complete infertility to heightened risks of miscarriage and adverse pregnancy outcomes like gestational hypertension and diabetes, PCOS also poses significant metabolic risks.^[2,3]^ These include but are not limited to endothelial dysfunction, hyperlipidemia, central obesity, dysregulated glucose homeostasis, and an elevated risk of cardiovascular diseases. Such extensive clinical ramifications render PCOS a considerable burden on both the reproductive health and overall quality of life of affected individuals.^[4]^

Despite extensive research into its pathogenesis, PCOS remains a complex enigma within the realm of reproductive endocrinology. Initial studies have predominantly focused on genetic predisposition, immunological factors, and androgenic exposure, yet these variables fall short of elucidating the disease’s multifaceted characteristics.^[2]^ Given this backdrop, it is imperative to understand the underlying mechanisms that contribute to PCOS, which is of paramount importance to the journal’s readership—comprising clinicians, endocrinologists, and researchers keen on progressive interventions.^[5]^ The advent of the Human Gut Metagenomic Project has shifted scientific focus towards the intricate interplay between gut microbiota and human health.^[6]^ This has led to emerging research highlighting a vital link between gut dysbiosis and the evolution of PCOS as a complex metabolic syndrome (MS).^[7]^

It is evident that despite strides in research, significant knowledge gaps exist in discerning the entirety of the pathogenesis of PCOS. The connection between gut dysbiosis and PCOS symbolizes one of these critical evidence gaps, thus accentuating the article’s importance for the journal’s readership.^[8]^ Extant studies indicate that females afflicted with PCOS often exhibit a distinct gut microbiota composition. Such microbial dysbiosis has been implicated in key pathological features of PCOS, including hyperandrogenism (HA), insulin resistance (IR), chronic inflammation, and metabolic irregularities.^[9]^ Furthermore, the correlation between gut dysbiosis and PCOS adds a new dimension to the disease understanding, thereby emphasizing the relevance and urgency of this review for researchers and clinicians alike.^[10–17]^ Consequently, the quest for a comprehensive understanding of its underlying pathogenesis and identification of innovative therapeutic strategies takes center stage in contemporary PCOS research and prevention, particularly in China. This review aims to elucidate the role of gut dysbiosis in the pathogenesis of PCOS, offering a novel lens through which to explore the disease’s pathology and thereby identify emerging avenues for targeted clinical interventions.

Given the emerging evidence and existing gaps in our understanding, this review has the following concrete aims: (1) To delineate the role of gut microbiota dysbiosis in the onset and progression of PCOS. (2) To elucidate the mechanistic links between gut dysbiosis and the key pathological features of PCOS. (3) To identify potential therapeutic targets within the gut microbiota and their implications for innovative interventions in PCOS management.

## 2. Overview of human gut microbiota: complexity, functions, and clinical implications

The human gut microbiota encompasses an incredibly diverse assembly of microorganisms, with estimates ranging from 10^13^ to 10^14^ individual cells, constituting more than 1000 distinct species and over 7000 strains.^[18,19]^ Genomically, the gut microbiota contains an estimated 150 times the genetic information found in the entire human genome.^[18]^ This microbial consortium comprises predominantly bacteria—specifically anaerobic bacteria—but it is not limited to them. It also consists of viruses, bacteriophages, archaea, and fungi, rendering it a multifaceted microbial ecosystem.^[18]^ The gut microbiota serves pivotal functions in host physiology. These functions encompass participation in immune modulation, safeguarding the integrity of the intestinal epithelial barrier, and facilitating the production of bioactive molecules like short-chain fatty acids (SCFAs).^[20]^ Intriguingly, the gut microbiota has recently been reconceptualized as a “second genome,” given its indispensable role in shaping and maintaining host physiology in a complex and mutualistic relationship with the human body.

Emerging evidence indicates that gut dysbiosis, or the imbalance of microbial communities, is associated with a myriad of conditions, including but not limited to metabolic, autoimmune, neurological, and cardiovascular diseases.^[20]^ In healthy individuals, inter-individual variations in microbiota composition have been implicated in susceptibility to a multitude of disorders.^[21]^ The composition of the gut microbiota is subject to alteration by a plethora of factors, such as genetic predisposition, maternal microbiota exposure during the intrauterine period, modes of childbirth and infant feeding, antibiotic administration, and environmental conditions.^[22]^ The implications of gut microbiota extend to conditions like PCOS, which is characterized by imbalances in sex hormones, IR, polycystic ovarian morphology, and chronic subclinical inflammation. The gut microbiota contributes to the pathology of PCOS through various mechanisms such as endotoxemia, SCFA metabolism, bile acid cycling, abnormal secretion of gut-brain peptides, and other processes that influence HA, IR, chronic inflammation, MS, and the gut-brain axis.^[23]^ The gut microbiota is thus implicated in follicular development, sex hormone functionality, and metabolic substrate concentrations.

### 2.1. Dysbiosis of gut microbiota and hyperandrogenemia in PCOS

HA is a cardinal feature of PCOS and constitutes a core pathological marker of the condition. Elevated androgen levels in the bloodstream are predominantly responsible for clinical manifestations such as hirsutism, acne, and anovulatory cycles. The connection between gut microbiota and sex hormones has primarily focused on assessing the impact on serum testosterone concentrations. Retrospective studies have revealed that women afflicted with PCOS exhibit diminished α-diversity of gut microbiota, which negatively correlates with serum testosterone levels, markers of HA, and hirsutism.^[14]^ Adolescents suffering from obesity and PCOS show altered gut microbiomes compared to non-PCOS counterparts; these alterations are also associated with metabolic aberrations and fluctuations in testosterone markers.^[24]^

In rodent models, the integrity of gut microbiota appears intrinsically linked to the homeostasis of sex hormones. Prepartum exposure to androgens has been associated with specific fecal microbiota profiles, characterized by increased bacterial populations involved in steroid hormone biosynthesis.^[25]^ Additionally, rats with dehydroepiandrosterone-induced PCOS have demonstrated gut microbiota dysbiosis; transplantation of such microbiota into healthy rats precipitated metabolic and endocrine dysfunctions mimicking PCOS.^[24,26]^ Subsequent investigations have highlighted that androgen-induced gut microbiota dysbiosis aggravates metabolic and endocrine imbalances in PCOS.^[27]^ Specific abnormalities in the gut microbiota of PCOS-affected women have been documented, including an elevated abundance of Bacteroidetaceae, Raoultella, and Prevotella, which correlates positively with androgen levels.^[10,13]^ Moreover, increased levels of Candida have been associated with circulating androstenedione.^[28,29]^ Lactobacilli have been linked positively to estradiol and estrone levels, and their transplantation has been shown to ameliorate the PCOS phenotype while reducing androgen biosynthesis.^[30]^

While the interplay between androgens and microbiota is increasingly acknowledged, existing studies have not delved sufficiently into the underlying mechanistic correlations. Various gut microbiota can produce enzymes responsible for the metabolism and conversion of androgens. Consequently, high levels of androgens may instigate dysbiosis in the host’s gut microbiota, impacting the pathophysiology and course of PCOS.^[31]^ The precise causal relationship between HA and gut microbiota dysbiosis remains nebulous. Future research endeavors should prioritize mechanistic studies to provide a robust theoretical framework and targeted therapeutic objectives for diagnosing and treating HA in PCOS.

### 2.2. Gut microbiota dysbiosis and IR in PCOS

Among women diagnosed with PCOS, approximately 25% to 70% present with varying degrees of IR.^[31]^ IR is increasingly recognized as a primary pathological driver in the development and progression of PCOS-related reproductive dysfunction.^[32]^ Specifically, the confluence of IR and compensatory hyperinsulinemia elicits an array of biological responses including perturbed sex hormone homeostasis, the perpetuation of chronic inflammatory states, and an increased susceptibility to metabolic disorders, cumulatively contributing to the suboptimal development of ovarian follicles.^[33]^ These etiological factors collectively precipitate a self-reinforcing pathogenic loop, complicating the clinical management of PCOS. Emerging research delineates a gut microbiota imbalance in individuals with PCOS.^[34]^ Despite the growing corpus of evidence, the literature remains deficient in elucidating the mechanistic underpinnings of how gut microbiota dysbiosis regulates the genesis and modulation of IR and its subsequent relationship with PCOS.^[35]^ Researchers have posited multifaceted pathways involving IR, gut microbiota, and PCOS, highlighting the complex interplay between these biological systems.^[36]^ Tremellen and colleagues^[37]^ proffered the hypothesis of “intestinal barrier endotoxemia inflammation,” elucidating its centrality in the pathophysiological landscape of PCOS.

Characterized by intestinal dysbiosis, degradation of the intestinal mucosal barrier, and augmented intestinal permeability, PCOS-affected women exhibit elevated indices of endotoxemia when compared to healthy counterparts.^[21]^ Lipopolysaccharides (LPS), major constituents of the cellular membrane of gram-negative bacteria such as Bacteroides and *Escherichia*, exert endotoxin effects pivotal to early-phase inflammation and an array of metabolic diseases. Perturbations in the gut microbiota facilitate LPS translocation into the systemic circulation,^[37]^ instigating endotoxemia.

Upon systemic dissemination, LPS is recognized and sequestered by Toll-like receptor 4 on the surface of immune cells through the intermediary involvement of LPS-binding protein, CD14, and myeloid differentiation factor-2. This binding event activates an intricate cascade of intracellular signaling pathways, culminating in the upregulation of pro-inflammatory cytokines, notably tumor necrosis factor α, and interleukin-6. These cytokines contribute to the state of chronic subclinical inflammation, reduced insulin sensitivity, elevated serum insulin levels, and, ultimately, the establishment of IR.^[38]^

Furthermore, LPS-mediated activation of kinases like c-Jun N-terminal kinase and inhibitor of nuclear factor-κB kinase leads to serine phosphorylation of insulin receptor substrate-1, thereby impairing insulin signaling pathways and exacerbating IR.^[38]^ Gastrointestinal hormones serve as another mechanistic conduit linking gut microbiota to IR. Altered gut microbiota composition influences the secretion of intestinal peptides, thereby modulating IR and hyperinsulinemia.^[39,40]^ Studies involving murine models, where fecal matter from PCOS patients was transplanted, demonstrated the emergence of IR within 10 weeks posttransplantation.^[41]^ Therapeutic administration of chenodeoxycholic acid in these murine models led to improved fasting and average blood glucose levels, suggesting that gut microbiota may ameliorate IR and modulate glucose metabolic disorders in PCOS via the Bacteroides-bile acid-intestinal farnesoid X receptor signaling axis.^[41]^ While IR is a substantiated correlate of gut microbiota dysfunction in the context of PCOS, the mechanistic nexus warranting this association remains to be fully characterized, underlining the imperative for focused, future investigations.

### 2.3. Gut microbiota dysbiosis and its influence on chronic inflammation in PCOS

Chronic inflammation serves as an integral modulator in the pathophysiological landscape of PCOS and represents a salient clinical feature in affected individuals. A plethora of both domestic and international studies have substantiated that individual with PCOS manifest elevated serum concentrations of pro-inflammatory markers, notably C-reactive protein and tumor necrosis factor α.^[42]^ These inflammatory mediators may exert a direct influence on the hypothalamic-pituitary-gonadal axis, thereby modulating follicular development, maturation, and subsequent ovulation in PCOS patients.^[43]^ However, the etiological underpinning of this elevated inflammatory state remains elusive. Significantly, the dysbiotic gut microbiota emerges as a critical mediator in establishing the pro-inflammatory milieu characteristic of both IR and PCOS. The interplay between inflammatory signaling cascades and insulin signaling pathways suggests that inflammation, IR, and obesity might converge principally due to “gut-induced endotoxemia.”^[44]^ Under conditions of gut dysbiosis, the tight junction proteins integral to the intestinal epithelial barrier experience downregulation, thereby undermining its structural integrity. This perturbation facilitates the systemic translocation of LPS, potent endotoxin elements found in the cell wall of gram-negative enteric bacteria.

Upon entry into the bloodstream, LPS interact with the CD14-Toll-like receptor 44 complex on the membrane of innate immune cells. This interaction sets in motion various inflammation-associated signaling pathways and culminates in the upregulation of pro-inflammatory cytokines, instigating a state of chronic inflammation. Furthermore, LPS can modulate serine phosphorylation of the insulin receptor substrate-1 and interfere with the canonical phosphorylation process of protein kinase B, via signaling mechanisms encompassing nuclear factor-κB, thereby contributing to IR.^[45]^ The degree of gut microbiota dysbiosis as precipitated by chronic inflammation may serve as a critical mechanistic element in the pathogenesis of PCOS. Elevated levels of circulating LPS instigate the upregulation of inflammatory mediators, ultimately exacerbating IR and further complicating the HA and metabolic aberrations associated with PCOS, while simultaneously impeding normal follicular development.

### 2.4. Interplay between gut microbiota dysbiosis and metabolic syndrome in PCOS

MS is a multifaceted pathological condition characterized by dysregulation in the metabolism of carbohydrates, lipids, proteins, and other bioactive molecules. Clinically, it manifests as hyperglycemia, hyperlipidemia, hypertension, and obesity. A noteworthy intersection exists between the metabolic consequences of PCOS and MS, with MS being disproportionately prevalent among individuals with PCOS as compared to the general population.^[46,47]^ Epidemiological surveys suggest that approximately 43.89% of patients diagnosed with PCOS also exhibit features of MS.^[48]^ Dietary factors, specifically a high-fat diet, are significant contributors to obesity and consequent MS. High-fat diets induce alterations in gut microbiota composition and intestinal permeability, thus elevating LPS production by gram-negative bacteria and attenuating the production of SCFAs. Emerging evidence implicates the SCFA metabolic pathway as a pivotal factor in the pathogenesis of PCOS.^[48]^ SCFAs, predominantly acetate, propionate, and butyrate, serve as crucial metabolic regulators, influencing glucose homeostasis, insulin sensitivity, anti-inflammatory actions, and immune regulation. Fecal analyses from PCOS patients indicate decreased levels of SCFAs,^[49]^ likely attributable to diminished populations of beneficial microbiota. SCFAs interact with G protein-coupled receptors (GPCR)43 and GPCR41 in various cellular entities, enhancing the production of glucagon-like peptide-1 (GLP-1) and peptide YY, thus facilitating insulin secretion and anti-inflammatory responses.^[49–51]^

Furthermore, SCFAs also bind to GPCR119 in enterochromaffin cells and pancreatic β cells. Agonism of GPCR119 triggers GLP-1 secretion, augments pancreatic β cell functionality, and enhances insulin production, thereby ameliorating hyperglycemia.^[52]^ The SCFA deficiency noted in PCOS patients thus exerts a detrimental impact on metabolic homeostasis through its influence on carbohydrate metabolism, pro-inflammatory effects, and obesity etiology. Additionally, branched-chain amino acids have emerged as harmful microbial-regulated metabolites that can influence insulin sensitivity.^[53]^ Previous study^[34]^ using murine models simulating PCOS pathology found that both obesity and long-term high-fat diets result in varying degrees of IR, presumably via branched-chain amino acid-mediated alterations in glucose metabolism or the induction of inflammation. In summation, a tangible link exists between the metabolic dysfunctions associated with carbohydrate and lipid metabolism and variations in gut microbiota. The gut microbiome modulates diverse metabolic pathways, including energy absorption, intestinal motility, and lipid and carbohydrate metabolism, thereby playing an integral role in the progression and exacerbation of MS.^[54]^

### 2.5. Role of gut microbiota in the progression of PCOS via the gut-brain axis

Emerging evidence underscores the importance of both the gut-brain axis and the hypothalamic-pituitary-ovarian axis in the complex pathogenesis of PCOS. The gut-brain axis serves as an intricate bidirectional communication network that intimately links the gastrointestinal and central nervous systems. Through diverse mechanisms including the vagus nerve pathway and the secretion of hormones and neurotransmitters, the gut microbiota plays an integral role in this complex information exchange system.^[55]^ Aberrations in the gut microbiota have been implicated in exacerbating PCOS via their influence on the gut-brain axis. Specifically, alterations in the microbiota composition and their metabolite profiles can trigger an array of physiological changes, including the secretion of gut-brain peptides, inflammation, IR, and hyperinsulinemia. Intestinal bacteria generate SCFAs such as acetate, propionate, and butyrate, which modulate the secretion of gut-brain peptides, including GLP-1, ghrelin, and peptide YY. Through GPCR43, SCFAs activate the mammalian target of rapamycin/signal transducer and activator of transcription signaling pathway, thereby regulating the synthesis of these peptides.^[56]^

Importantly, GLP-1 has been recognized as a potent stimulator of gonadotropin-releasing hormone (GnRH) neurons, affecting kisspeptin and gamma-aminobutyric acid (GABA)ergic neurons. It modulates the secretion of GnRH as well as luteinizing hormone, subsequently influencing the manifestation of PCOS.^[57]^ Ghrelin, another gut-derived peptide, has the potential to regulate the hypothalamic-pituitary axis and the secretion of luteinizing hormone, thereby ameliorating the reproductive dysfunction commonly observed in PCOS patients. Additionally, gut microbiota synthesizes neurotransmitters such as dopamine, norepinephrine, serotonin, and GABA, which further influence PCOS progression. Particularly, GABA activates GnRH neurons, thereby affecting the frequency and amplitude of GnRH pulses.^[58]^ Other microbiota-derived metabolites, like bile acids, also serve as potent regulators of GnRH neuron function.^[57]^ SCFAs specifically induce the secretion of GLP-1 via receptors GPR-41/43 present on intestinal L cells.^[59]^ In conclusion, the interplay between gut microbiota, gut-brain axis mediators, and PCOS presents a complex and not yet fully elucidated relationship.^[60–62]^ Further studies are warranted to fully comprehend the mechanistic underpinnings of how the gut microbiota contribute to the development and progression of PCOS through the gut-brain axis.

## 3. Summary and future directions

PCOS is a multifactorial disorder influenced by genetic predispositions, neuroendocrine imbalances, metabolic dysfunction, environmental factors, and lifestyle considerations. Despite extensive research, the pathogenesis of PCOS remains incompletely understood. Advancements in next-generation sequencing technologies and metabolomics have paved the way for increasing focus on the role of gut microbiota in the etiology and progression of PCOS. Dysbiosis in the gut microbiota exerts a significant regulatory influence on HA, IR, chronic inflammation, and metabolic disturbances, thereby contributing to the onset and exacerbation of PCOS via multiple pathways.

While the importance of the gut microbiota in PCOS has been established, there is a scarcity of comprehensive studies elucidating the underlying mechanisms. Specifically, the understanding of microbiota–hormone interactions and their role in endocrine and metabolic dysregulation related to PCOS is still in its infancy. Identifying and evaluating the functional bacterial strains associated with the disease could offer new insights into its pathogenesis. This could, in turn, pave the way for targeted microbiome modulation as a therapeutic strategy.

Therefore, urgent issues that warrant attention include the exploration of the etiological underpinnings and mechanisms by which gut microbiota influence hormone-mediated disorders in PCOS. Further research is needed to pinpoint specific bacterial spectra that are implicated in the initiation and progression of the disease. These future investigations hold the potential for establishing new treatment paradigms and preventive strategies aimed at ameliorating the pathological processes underpinning PCOS on a fundamental level.

## Author contributions

**Investigation:** Hua Guo, Jing Luo.

**Supervision:** Hanmei Lin.

**Validation:** Jing Luo.

**Writing – original draft:** Hua Guo.

**Writing – review & editing:** Hanmei Lin.
